# Identification of *M. tuberculosis* Rv3441c and *M. smegmatis* MSMEG_1556 and Essentiality of *M. smegmatis* MSMEG_1556

**DOI:** 10.1371/journal.pone.0042769

**Published:** 2012-08-08

**Authors:** Shuang Li, Jian Kang, Wendan Yu, Yan Zhou, Wenli Zhang, Yi Xin, Yufang Ma

**Affiliations:** 1 Department of Biochemistry and Molecular Biology, Dalian Medical Universtiy, Dalian, Liaoning, China; 2 Department of Biotechnology, Dalian Medical University, Dalian, Liaoning, China; 3 Liaoning Provincial Core Lab of Glycobiology and Glycoengineering, Dalian, Liaoning, China; University of Padova, Italy

## Abstract

The normal growth of mycobacteria attributes to the integrity of cell wall core which consists of peptidoglycan (PG), arabinogalactan (AG) and mycolic acids. N-acetyl glucosamine (GlcNAc) is an essential component in both PG and AG of mycobacterial cell wall. The biosynthetic pathway for UDP-N-acetylglucosamine (UDP-GlcNAc), as a sugar donor of GlcNAc, is different in prokaryotes and eukaryotes. The conversion of glucosamine-6-phosphate to glucosamine-1-phosphate, which is catalyzed by phosphoglucosamine mutase (GlmM), is unique to prokaryotes. Bioinformatic analysis showed that Msm MSMEG_1556 and Mtb Rv3441c are homologous to Ec GlmM. In this study, soluble Msm MSMEG_1556 protein and Mtb Rv3441c protein were expressed in *E. coli* BL21(DE3) and their phosphoglucosamine mutase activity were detected. In order to further investigate the essentiality of MSMEG_1556 for the growth of *M. smegmatis,* we generated a conditional MSMEG_1556 knockout mutant, which harbored thermo-sensitive rescue plasmid carrying Mtb Rv3441c. As the rescue plasmid was unable to complement MSMEG_1556 deficiency at 42°C, MSMEG_1556 knockout mutant did not grow. The dramatic morphological changes of MSMEG_1556 knockout mutant after temperature shift from 30°C to 42°C have been observed by scanning electron microscope. These results demonstrated that MSMEG_1556 is essential for growth of *M. smegmatis*. This study provided evidence that GlmM enzyme could be as a potential target for developing anti-tuberculosis drugs.

## Introduction

Nowadays, two millions deaths each year (2% increased incidence) caused by *Mycobacterium tuberculosis* has been considered to be a major public health threat [1]. However, vaccine failed to provide protective immunity and any efforts to control tuberculosis were compromised as they evolved into stronger, more drug-resistant forms [2], [3], [4], [5]. As we know, the cell wall of all Mycobacterium species is very waxy, hydrophobic, and thicker than other bacteria, the low permeability and resistance of cell wall substantially contributes to the defense of adverse factors [6], [7]. Thereby, the enzymes involved in the metabolic pathways of the cell wall are potential excellent targets for new anti-tuberculosis drugs [8], [9].

**Figure 1:**
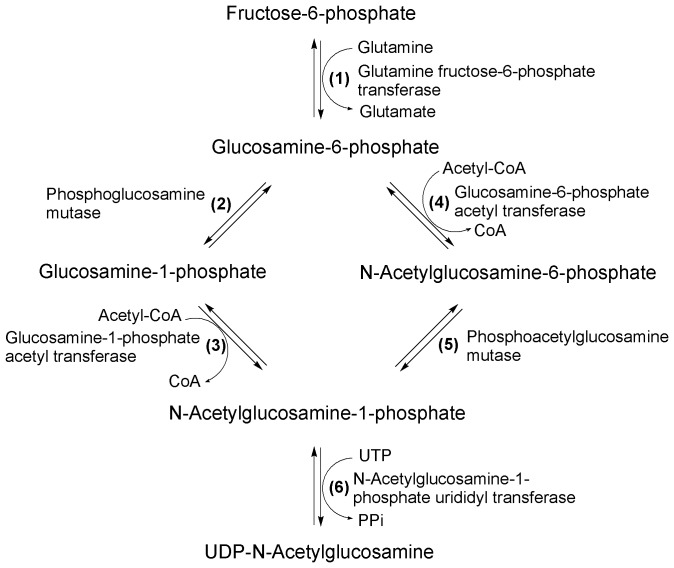
Biosynthetic pathway of UDP-GlcNAc in prokaryotes and eukaryotes. The biosynthetic pathway of UDP-GlcNAc in prokaryotes and eukaryotes were different. In prokaryotes, UDP-GlcNAc was biosynthesized through reactions (1), (2), (3) and (6); In eukaryotes, UDP-GlcNAc was formed through reactions (1), (4), (5) and (6). The reactions catalyzed by phosphoglucosamine mutase (GlmM) and glucosamine-1-phosphate acetyl transferase (GlmU) were unique to prokaryotes.

The mycobacterial cell wall consists of the mycolate and peptidoglycan (PG) layer held together by arabinogalactan (AG) layer [10], [11]. AG is attached to the muramic acid residue of the PG through a disaccharide linker (α-L-rhamnosyl-α-D-N-acetylglucosaminosyl-1-phosphate), and the glycan of PG is a disaccharide repeat unit (N-acetylmuramic acid-N-acetyl glucosamine). UDP-N-acetylglucosamine (UDP-GlcNAc) is an important precursor for the synthesis of PG layer, and also a direct glycosyl donor for disaccharide linker, therefore, it perhaps plays a vital role in mycobacterial growth [12], [13], [14]. Three enzymes glutamine fructose-6-phosphate transferase (GlmS), phosphoglucosamine mutase (GlmM), glucosamine-1-phosphate acetyl transferase/N-Acetylglucosamine-1-phosphate urididyl transferase (GlmU) involve in the metabolic pathway of UDP-GlcNAc in *E. coli* [13], [15], [16], [17], [18]. It is noteworthy that reactions catalyzed by GlmM and GlmU are unique to prokaryotes (Fig. 1). The function of Msm GlmU and Mtb GlmU had been identified and the GlmU had been confirmed being essential for growth of *M. smegmatis* and *M. tuberculosis* [19], [20]. Therefore, GlmM may also be essential for mycobacteria and could be used as a potential target of anti-tuberculosis drug.

The bioinformatics analysis data showed that Msm MSMEG_1556 and Mtb Rv3441c are homologous to Ec GlmM. We proposed Msm MSMEG_1556 protein and Mtb Rv3441c protein catalyzing the same reaction in biosynthetic pathway of UDP-GlcNAc as in *E. coli*. In this study, we identified that both Msm MSMEG_1556 and Mtb Rv3441c protein had the phosphoglucosamine mutase activity. The essentiality of MSMEG_1556 for mycobacteria was determined by observing growth curve, colony forming unit (CFU) and morphology of MSMEG_1556 knockout mutant. In addition, dinitrothiocyanobenzene (DNTB) chromogenic method [20], [21] in microtiter plate for the measurement of phosphoglucosamine mutases activity was described.

## Materials and Methods

### Bacterial strains, plasmids, and culture conditions

The bacterial strains and plasmids used in this study were listed in Table S1. *E.coli* DH5α, NovaBlue and BL21(DE3) cells were grown in Luria-Bertani (LB) medium at 37°C routinely. *M. smegmatis* mc^2^155 strain was grown in LB broth containing 0.05% Tween 80 or on LB agar at 37°C routinely. *M. smegmatis* mc^2^155 was used for cloning MSMEG_1556 gene with its upstream region and constructing a conditional *M. smegmatis* MSMEG_1556 gene knockout strain by allelic exchange recombinant experiments. Sucrose was added to the LB agar at final concentration of 10% when required. Antibiotics were added at the following final concentrations: ampicillin (Amp), 100 µg/ml; gentamicin (Gen), 5 µg/ml for *E. coli*; kanamycin (Kan), 50 µg/ml for *E. coli* and 25 µg/ml for *M. smegmatis*; streptomycin (Str), 25 µg/ml for *E. coli* and 12.5 µg/ml for *M. smegmatis*.

### Sequence alignment of Ec GlmM, Msm MSMEG_1556 and Mtb Rv3441c

The amino acid sequences of Ec GlmM protein, Msm MSMEG_1556 protein and Mtb Rv3441c protein were aligned by using Multalin online tool (http://multalin.toulouse.inra.fr/multalin/) [22].

### Expression and purification of Ec GlmM protein, Msm MSMEG_1556 protein, Mtb Rv3441c protein, and Mtb GlmU protein

The genomic DNA of *E. coli* BL21(DE3) was prepared as described previously [23], with modification. Ec *glmM* gene was amplified from *E. coli* BL21(DE3) genomic DNA using the Ec glmM1 and Ec glmM2 primers (Table 1) and was cloned into pJET1.2/blunt vector to generate a plasmid pKJ3 (pJET-Ec *glmM*) (Table S1). The genomic DNA of *M. semgmatis* mc^2^155 was prepared as described from 20 ml culture [11]. The Msm MSMEG_1556 was amplified from *M. smegmatis* mc^2^155 genomic DNA by PCR using Msm glmM1 and Msm glmM2 primers (Table 1) and was cloned into pJET1.2/blunt vector to generate pKJ1 (pJET-Msm MSMEG_1556) plasmid (Table S1). The Mtb Rv3441c gene was amplified from H37Rv genomic DNA (supplied by Colorado State University via an NIH contract) by using Mtb glmM1 and Mtb glmM2 primers (Table 1), and was cloned into pMD18-T vector to generate pLS1 (pMD18-Mtb Rv3441c) plasmid (Table S1). After confirmation by DNA sequencing, the Ec *glmM* was ligated into the NcoI and BamHI sites of pET16b, resulting in pKJ4 (pET16b-Ec *glmM*) (Table S1). The Msm MSMEG_1556 was ligated into the NdeI and EcoRI sites of pCold II, resulting in pKJ2 (pCold II -MSMEG_1556) (Table S1), and the Mtb Rv3441c was ligated into the NdeI and BamHI sites of pCold II yielding pLS2 (pCold II-Mtb Rv3441c) plasmid (Table S1). pKJ4, pKJ2 and pLS2 were transformed to *E. coli* BL21(DE3) for overexpression of Ec GlmM protein, Msm MSMEG_1556 protein and Mtb Rv3441c protein, respectively.

**Table 1:** Primers used in this study.

Primers	Primer sequences (5′→3′)
Ec glmM1	GCCCATGGTGATGAGTAATCGTAAATATTTCG (underlined is NcoI site)
Ec glmM2	TTGGATCCTTAATGATGATGATGATGATGAACGGCTTTTACTGCATCGGCG (underlined is BamHI site)
Msm glmM1	TTCATATGGCTCGACTGTTCGGCAC (underlined is NdeI site)
Msm glmM2	CCGAATTCTATCCCTGCAGACTCAC (underlined is EcoRI site)
Mtb glmM1	GGCGCATATGGGTCGACTGTTTGGCAC (underlined is NdeI site)
Mtb glmM2	TAATGGATCCTCAGCGCGCGGTGCTCACCG (underlined is BamHI site)
Msm glmM3	GACTAGTGGTGTCCTCGAAGACGTGCCCATCGG (underlined is SpeI site)
Msm glmM4	TGCGGCCGCCTATCCCTGCAGACTCACGGATTC (underlined is NotI site)
Msm glmM5	ACTGGGCACCGCAGGTGTC
Msm glmM6	GTTCGGCGACTATCCCTGC

*E. coli* BL21(DE3) strains carrying different expression plasmids were grown at 37°C in 300 ml LB broth containing Amp. When OD_600_ of the culture reached to 0.5, BL21(DE3)/pKJ4 culture was induced by 0.4 mM isopropyl-D-thiogalactopyranoside (IPTG) at 37°C for 3 hours; BL21(DE3)/pKJ2 and BL21(DE3)/pLS2 was transferred to a 15°C-incubator and induced by 1 mM IPTG for 24 hours. The cells were then harvested and resuspended in 8 ml lysis buffer (20 mM Tris-HCl, pH 8.0, 500 mM NaCl and 20% glycerol) with 1 mM phenylmethyl-sulphonyl fluoride (PMSF) followed by sonication. After centrifugation at 20,000×g for 30 minutes, the resulting supernatant was loaded onto 1 ml Ni-NTA column (Qiagen) previously equilibrated with equilibrium buffer. The column was washed with 20 ml wash buffer (20 mM Tris-HCl, pH 8.0, 500 mM NaCl, 20% glycerol and 45 mM imidazole). The purified protein was eluted with 15 ml elute buffer (20 mM Tris-HCl, pH 8.0, 500 mM NaCl, 20% glycerol and 200 mM imidazole) with 1 mM PMSF, and the first 7 ml was collected for analyses of SDS-PAGE and Western blot as well as detection of phosphoglucosamine mutase activity.

The purified Ec GlmM protein, Msm MSMEG_1556 protein and Mtb Rv3441c protein were run on 12% SDS-PAGE and transferred to a nitrocellulose membrane (Pall Corp) in blotting buffer (20 mM Tris-base, 150 mM glycine and 20% methanol). The membrane was blocked with 1% BSA in TBST buffer (10 mM Tris–HCl, pH 8.0, 150 mM NaCl, 0.05% Tween 20) and incubated with (anti)-polyhistidine clone His-1 antibody (Sigma) at 1∶5000 dilution followed by washing with TBST. The membrane was incubated with antimouse-IgG-AP conjugate and the protein band was visualized in BCIP/NBT solution.

The Mtb GlmU protein was purified as described previously [19].

### Assaying phosphoglucosamine mutase of Ec GlmM protein, Msm MSMEG_1556 protein and Mtb Rv3441c protein

**Figure 2:**
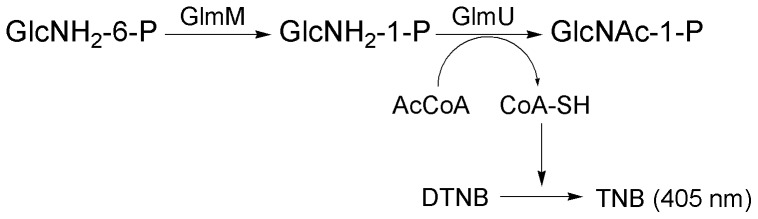
Assay of phosphoglucosamine mutase. Phosphoglucosamine mutase catalyzes the conversion of GlcNH_2_-6-P to GlcNH_2_-1-P. GlcNH_2_-1-P and acetyl CoA were catalyzed by glucosamine-1-phosphate acetyltransferase activity of GlmU to produce GlcNAc-1-P and CoA-SH which was detected by DTNB. TNB, the product generated from the reaction of CoA-SH and DTNB, could be monitored at 405 nm.

The phosphoglucosamine mutase activity of Ec GlmM protein, Msm MSMEG_1556 protein and Mtb Rv3441c protein were determined by coupled assay, in which the GlcNH_2_-1-P converted from GlcNH_2_-6-P by the mutase was quantitatively converted into UDP-GlcNAc in the presence of purified GlmU enzyme [24]. The by-product CoA-SH from the reaction catalyzed by GlmU acetyltransferase activity was then measured by a colorimetric assay coupled with 5, 5′-dithio-bis-(2-nitrobenzoic acid) (DTNB) (Fig. 2). The reaction mixture of 50 µl contained 50 mM Tris-HCl, pH8.0, 2.5 mM MgCl_2_, 1 mM GlcNH_2_-6-P, 0.6 mM acetyl CoA, 0.7 mM Glc-1, 6-diP, purified Mtb GlmU protein (1 µg) and purified Ec GlmM protein (0.625 µg), Msm MSMEG_1556 protein (0.625 µg) or Mtb Rv3441c protein (0.625 µg) in a 96-well microtiter plate. A control without GlmM protein was used to correct the trial errors from the reaction of GlmU protein with GlcNH_2_-6-P. The reaction was incubated at 37°C for 30 min and terminated by adding 50 µl of stop solution containing 50 mM Tris-HCl, pH 7.5, 6 M guanidine hydrochloride and then incubated for 10 min by the addition of 50 µl of Ellman's reagent solution containing 0.2 mM DTNB in buffer with 50 mM Tris-HCl, pH 7.5, and 1 mM EDTA. TNB, the product generated from the reaction of CoA-SH and DTNB, was monitored at 405 nm by Benchmark Plus plate reader (Thermo). To confirm product CoA-SH the reaction mixture was applied on a Nova-Pak C18 (3.9×150 mm, 4 µm) and CoA-SH was separated at a flow rate of 1 ml/min under phosphate buffer (pH 6.5)-methanol (95∶5) and monitored at 259 nm. One unit of specific enzyme activity was defined as one nmol CoA-SH produced by one mg protein per minute under the specific condition.

### Construction of conditional *M. smegmatis* MSMEG_1556 gene knockout strain

Msm MSMEG_1556 gene (1362 bp) with its upstream sequence (500 bp) was amplified from mc^2^155 genomic DNA by using Msm glmM3 and Msm glmM4 primers (Table 1). The PCR product (1862 bp) was purified and cloned into pMD18-T vector to yield pLS3 (pMD18-Msm MSMEG_1556) plasmid (Table S1). After confirmation by DNA sequencing, the *kan^R^* cassette from pUC4K was inserted to the XhoI site of pLS3, resulting in pLS4 (pMD18-Msm MSMEG_1556::*kan^R^*) plasmid (Table S1). pLS4 was digested by NotI and SpeI and the Msm MSMEG_1556::*kan^R^* fragment was cloned to the NotI and SpeI sites of pPR27-*xylE* to generate a conditional replication plasmid pLS5 (pPR27-Msm MSMEG_1556::*kan^R^*) (Table S1). pLS5 also harbors the counter-selectable marker *sacB* from *Bacillus subtilis* for use in selecting the double crossover event in the presence of sucrose.

Since both Mtb Rv3441c gene and Msm MSMEG_1556 gene were identified as *glmM* gene to encode phosphoglucosamine mutase, Mtb Rv3441c gene was used for construction of the rescue plasmid. pLS2 was digested by NdeI and BamHI and the Mtb Rv3441c gene was ligated to the NdeI and BamHI sites of pET23b-Phsp60, yielding pLS6 (pET23b-Phsp60-Mtb Rv3441c) plasmid. pLS6 was digested by XbaI and BamHI and Phsp60-Mtb Rv3441c fragment was cloned to the XbaI and BamHI sites of pCG76 to generate a rescue plasmid pLS7 (pCG76-Phsp60- Mtb Rv3441c) [25].

*M. smegmatis* mc^2^155 electro-competent cells were prepared as described [26]. The conditional replication plasmid pLS5 was electroporated into mc^2^155 competent cells by Electroporator 2510 (Eppendorf). The transformants were then grown on LB agar plates containing Kan and Gen at 30°C. One colony was incubated in LB broth containing 0.05% Tween 80, Kan and Gen at 30°C and the culture was then plated out onto an LB agar plate containing Kan and Gen followed by incubation at 42°C for 6 days. Since plasmid pLS5 was not able to replicate at 42°C, the MSMEG_1556::*kan^R^* in pLS5 made single crossover at upstream or downstream of the MSMEG_1556 locus in *M. smegmatis* genome of colonies grown at 42°C. *M. smegmatis* LS1 mutants (*M. smegmatis* mc^2^155 with pLS5 plasmid integrated into the MSMEG_1556 gene locus) (Table S1) through the first homologous recombination were selected by Southern blot [11].

The rescue plasmid pLS7 was electroporated into *M. smegmatis* LS1 electro-competent cells and the transformants were grown on an LB agar plate containing Kan, Gen and Str and grown at 30°C. One colony was incubated in LB broth containing 0.05% Tween 80, Kan, Gen and Str and grown at 30°C. The culture was then plated out onto LB agar plate containing Kan, Str and 10% sucrose. *M. smegmatis* LS2 (mcΔ1556::pLS7), a MSMEG_1556 gene knockout strain (Table S1) where Msm MSMEG_1556 gene was knocked out through the second homologues recombination was selected by using Southern blot. To confirm *M. smegmatis* LS2, the DNA fragment containing Msm MSMEG_1556::*kan^R^* and its flanking sequences at upstream and downstream of Msm MSMEG_1556 was amplified from *M. smegmatis* LS2 genome by using Msm glmM5 and Msm glmM6 primers (Table 1). The amplified PCR products of Msm MSMEG_1556::*kan^R^* from *M. smegmatis* LS2 and Msm MSMEG_1556 from wild type *M. smegmatis* mc^2^155 were distinguished by 1% agrose gel.

### Southern blot analysis

The MSMEG_1556 DNA probe was prepared by using DIG High Prime Labeling and Detection Starter Kit I (Roche). The pLS3 was digested by SpeI and NotI and Msm MSMEG_1556 was purified. One microliter of the purified Msm MSMEG_1556 was used as a template to generate probe. The genomic DNA isolated from different colonies was digested overnight by SmaI enzyme. The resulting DNA fragments were separated by a 0.8% agarose gel. The DNA was then transferred to Hybond-N^+^ membrane (GE Healthcare). DNA hybridization and detection of the probe were performed as recommended by Roche.

### Growth of *M. smegmatis* LS2 strain

The growth of *M. smegmatis* LS2 was determined by observing growth curve and CFU, respectively. To measure growth curve, *M. smegmatis* LS2 was inoculated into 5 ml of LB broth containing 0.05% Tween 80 and appropriate antibiotics, and the cells were incubated at 30°C and 42°C respectively. *M. smegmatis* mc^2^155 carrying the rescue plasmid pLS7 and wild type *M. smegmatis* mc^2^155 were used as controls. The OD_600_ of the cultures was monitored at interval of 24 h and the growth curves at both 30°C and 42°C were obtained.

To measure CFU, *M. smegmatis* LS2 was grown in LB medium containing 0.05% Tween 80 and Kan at 30°C until the OD_600_ reached 0.10, and the culture was transferred to a 42°C incubator. The culture grown at 30°C was as control. The OD_600_ was determined at the interval of 24 hours. For CFU determinations (taken at the same time points), dilutions were spread on LB agar plates containing Str and Kan. The plates were incubated at 30°C before CFU were counted.

### Expression of MSMEG_1556 protein in *M. smegmatis* LS2 strain

Western blot was used to analyze the expression levels of GlmM protein in *M. smegmatis* LS2. *M. smegmatis* LS2 was grown in 500 ml LB broth at 30°C, and then switched to 42°C when OD_600_ of the culture was 0.01. *M. smegmatis* LS2 kept growing at 30°C was as control. The culture after temperature shifting for 72 h was harvested and the cells were resuspended in 4 ml lysis buffer with 1 mM PMSF followed by sonication. After centrifugation at 20,000×g for 30 minutes, the resulting supernatant was collected for Western blot analyses. The protocol was followed as previously described but (anti)-MSMEG_1556 antibody (prepared in our lab, unpublished data) was used to instead of the (anti)-polyhistidine clone His-1 antibody.

### Morphology of *M. smegmatis* LS2 strain after shifting temperature from 30°C to 42°C

*M. smegmatis* LS2 was grown in 20 ml LB broth at 30°C, and then switched to 42°C when OD_600_ of the culture was 0.01. *M. smegmatis* LS2 kept growing at 30°C was as control. The cells were harvested after shifting temperature for 72 h, 120 h and 192 h, respectively. Scanning electron microscopy (SEM) samples were prepared as previously described [19]. Briefly, the cell pellet was washed three times in the 0.1 M Phosphate buffer (pH 7.4) and fixed with 2.5% glutaraldehyde followed by fixation with 1% OsO4. The cells were then dehydrated in a graded series of ethanol (20, 40, 60, 70, 80, 90, and 100%). After critical point dry the cells were applied to a silicon wafer slide and coated by gold. The cells were then observed by using a JSM-6360 at 15 kV of accelerating voltage.

## Results

### Msm MSMEG_1556 and Mtb Rv3441c were homologous to Ec GlmM

**Figure 3:**
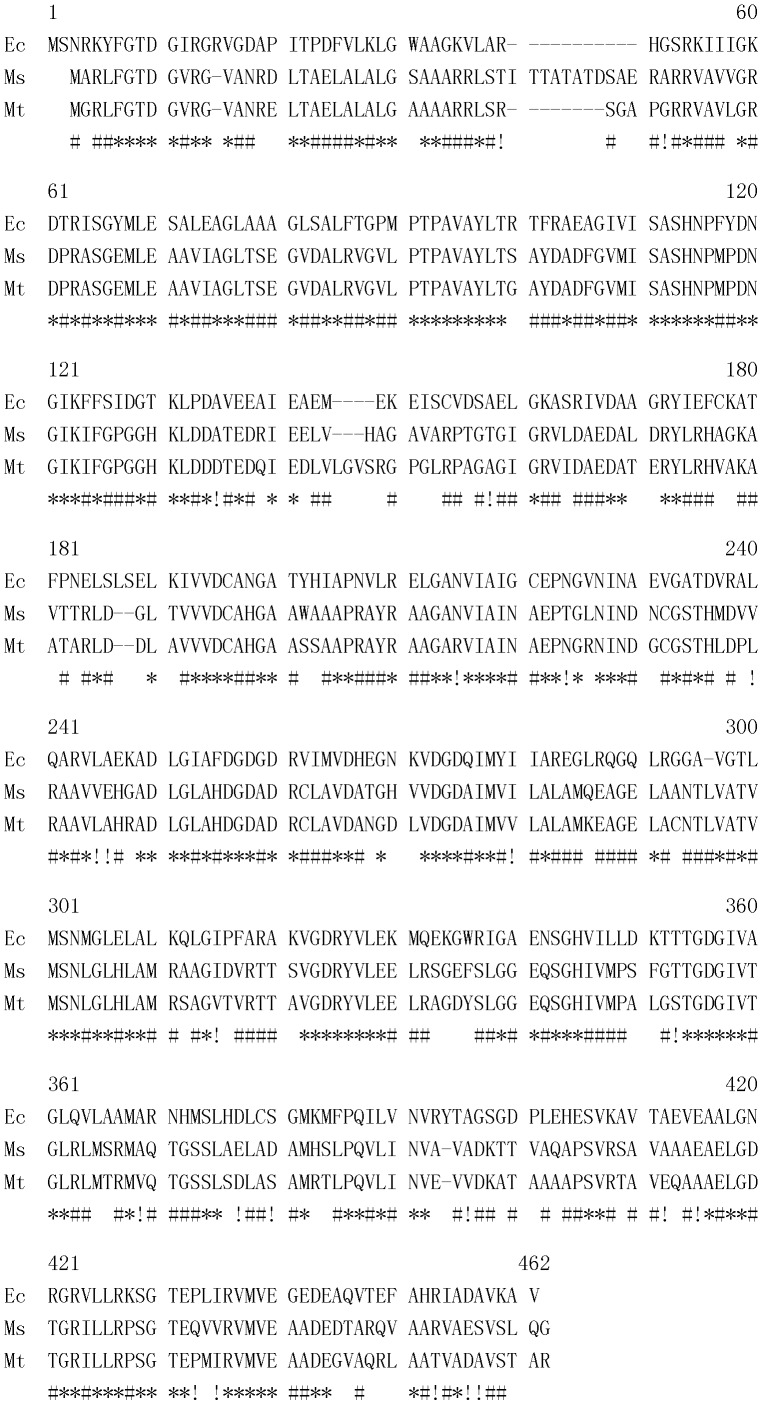
Sequence alignment of Ec GlmM, Msm MSMEG_1556 and Mtb Rv3441c. Msm MSMEG_1556 protein was 38% identical to Ec GlmM and Mtb Rv3441c protein was 41% identical to Ec GlmM. Mtb Rv3441c protein and Msm MSMEG_1556 protein had 79% identities. * indicates the homology between all three organisms, # indicates the homology between *M. tuberculosis* and *M. smegmatis*, and ! indicates the homology between *E. coli* and *M. smegmatis* or *E. coli* and *M. tuberculosis*.

The bioinformatics analysis data showed that Msm MSMEG_1556 protein was 38% identical to Ec GlmM and Mtb Rv3441c protein was 41% identical to Ec GlmM. Msm MSMEG_1556 protein and Mtb Rv3441c protein had 79% identities (Fig. 3). The results demostrated that Rv3441c is the ortholog of GlmM in *M. tuberculosis* and MSMEG_1556 is the ortholog of GlmM in *M. smegmatis*.

### Soluble Ec GlmM protein, Msm MSMEG_1556 protein and Mtb Rv3441c protein were overexpressed

**Figure 4:**
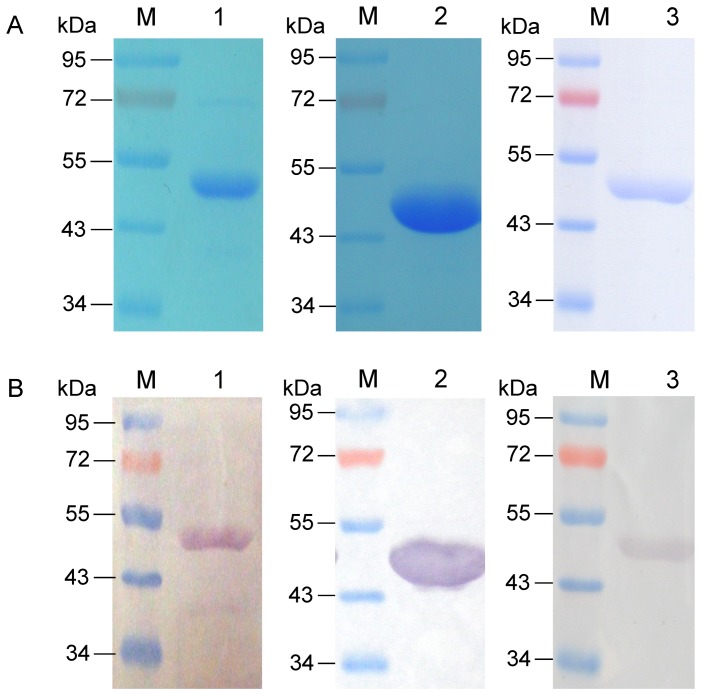
Analysis of the purified Ec GlmM protein, Msm MSMEG_1556 protein and Mtb Rv3441c protein by SDS-PAGE (A) and Western blot (B). A. SDS-PAGE analysis. M. PageRuler prestained protein ladder (Fermentas); lane 1. the purified Ec GlmM protein with an expected molecular weight of 48.99 kDa; lane 2. the purified Msm MSMEG_1556 protein with an expected molecular weight of 47.99 kDa; lane 3. the purified Mtb Rv3441c protein with an expected molecular weight of 47.31 kDa. B. Western blot analysis. M. PageRuler prestained protein ladder (Fermentas); lane 1. the purified Ec GlmM protein; lane 2. the purified Msm MSMEG_1556 protein; lane 3. the purified Mtb Rv3441c protein.

Soluble Ec GlmM protein, Msm MSMEG_1556 protein and Mtb Rv3441c protein were overexpressed in BL21(DE3) respectively and purified by Ni-NTA affinity chromatography. The purified proteins were analyzed by SDS-PAGE and Western blot. The results showed that purified Ec GlmM protein, Msm MSMEG_1556 protein and Mtb Rv3441c protein had the expected molecular weight of 48.99 kD, 47.99 kD, and 47.31 kD, respectively (Fig. 4).

### Msm MSMEG_1556 protein and Mtb Rv3441c protein had phosphoglucosamine mutase activity

The specific enzyme activity of Ec GlmM, which could be as a positive control, was 156 nmol·min^−1^·mg^−1^. The specific enzyme activity of Msm MSMEG_1556 protein and Mtb Rv3441c protein were 145 and 151 nmol·min^−1^·mg^−1^, respectively. The results demonstrated that both Msm MSMEG_1556 protein and Mtb Rv3441c protein had phosphoglucosamine mutase activity same as Ec GlmM protein (Table 2). In the reaction catalyzed by Mtb Rv3441c protein, the product CoA-SH was also detected by Nova-Pak C18 and its retention time was 9.1 min, which is consistent with CoA-SH standard (data not shown).

**Table 2:** Specific phosphoglucosamine mutase activity of GlmM proteins.

Proteins	Specific enzyme activity of (nmol·min^−1^·mg^−1^)
Purified Ec GlmM	156
Purified Msm MSMEG_1556	145
Purified Mtb Rv3441c	151

### Conditional *M. smegmatis* MSMEG_1556 gene knockout strain LS2 was constructed

**Figure 5:**
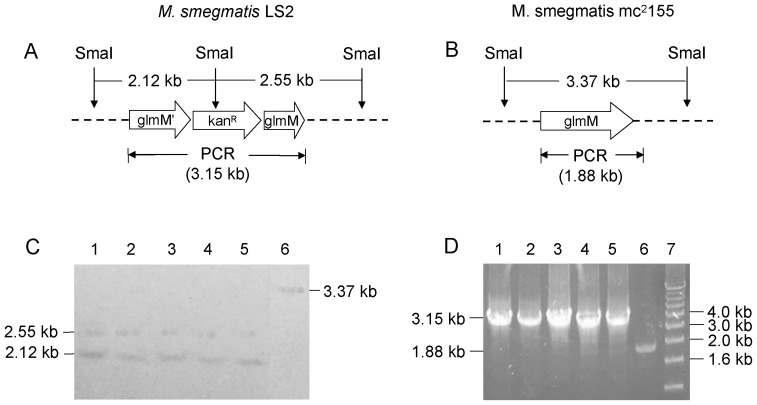
Southern blot and PCR analyses of *M. smegmatis* LS2 strains. The genomic DNA was digested overnight by SmaI enzyme. The resulting DNA fragments were separated by a 0.8% agarose gel. The DNA was then transferred to Hybond-N^+^ membrane and hybridized by MSMEG_1556 probe. A. The expected DNA fragments hybridized by MSMEG_1556 probe were 2.12 kb and 2.55 kb. The expected size of PCR product from *M. smegmatis* LS2 strain was 3.15 kb. B. The expected DNA fragments hybridized by MSMEG_1556 probe was 3.37 kb and the expected size of PCR product from *M. smegmatis* mc^2^155 strain was 1.88 kb. C. Confirmation of *M. smegmatis* LS2 strains by Southern blot. Lanes 1–5. *M. smegmatis* LS2 strains have the hybridized DNA bands of 2.12 kb and 2.55 kb; lane 6. wild type *M. smegmatis* mc^2^155 has the hybridized DNA band of 3.37 kb. D. Confirmation of *M. smegmatis* LS2 strains by PCR. Lanes 1–5. the PCR product (3.15 kb) from *M. smegmatis* LS2 strains; lane 6. the PCR product (1.88 kb) from wild type *M. smegmatis* mc^2^155.

Once conditional replication plasmid pLS5 was electroporated to *M. smegmatis* mc^2^155, the MSMEG_1556::*kan^R^* in pLS5 made single crossover at upstream or downstream of the MSMEG_1556 locus in *M. smegmatis* genome of colonies grown at 42°C, resulting in *M. smegmatis* LS1 mutants which integrated the MSMEG_1556::*kan^R^* at the MSMEG_1556 locus through the first homologous recombination. *M. smegmatis* LS1 mutants were selected by Southern blot (data not shown). The results suggested that MSMEG_1556 was an essential gene. When rescue plasmid pLS7 was electroporated to *M. smegmatis* LS1 mutant, the colonies grown on LB agar plate containing Kan, Str and Sucrose at 30°C had undergone the second homologous recombination, resulting in MSMEG_1556 gene knockout. Five *M. smegmatis* LS2 (MSMEG_1556 gene knockout) strains were selected by Southern blot. All five *M. smegmatis* LS2 strains showed the expected DNA fragments of 2.12 kb and 2.55 kb (Fig. 5A, 5C), whereas wild type *M. smegmatis* mc^2^155 showed an expected DNA fragment of 3.38 kb (Fig. 5B, 5C). The PCR result indicated that all five *M. smegmatis* LS2 strains had the expected PCR product of 3.15 kb, and wild type *M. smegmatis* mc^2^155 showed the expected PCR product of 1.88 kb (Fig. 5D).

### MSMEG_1556 gene was essential for mycobacterial growth

**Figure 6:**
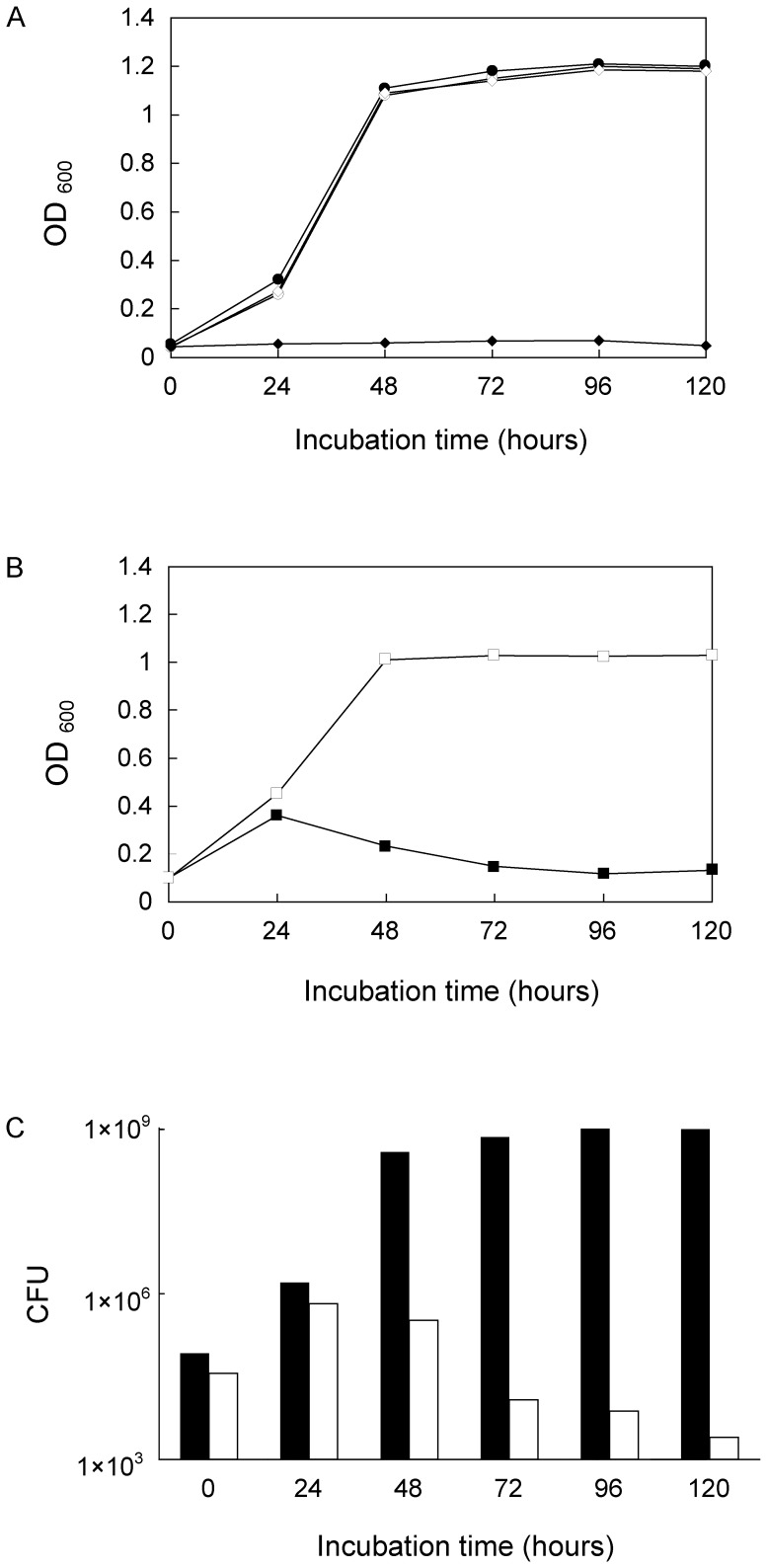
Growth curves and CFU of *M. smegmatis* LS2 strain. A. Growth curves of *M. smegmatis* LS2 and *M. smegmatis* mc^2^155 carrying pCG76 plasmid at 30°C and 42°C. The cultures were grown in LB medium at 30°C and 42°C, and growth was monitored by measuring the absorbance of the cultures at 600 nm. *M. smegmatis* mc^2^155 strains carrying pCG76 plasmid had similar growth patterns at 30°C and 42°C. *M. smegmatis* LS2 was only able to grow at 30°C but not at 42°C. (⋄) *M. smegmatis* LS2 at 30°C; (⧫) *M. smegmatis* LS2 at 42°C; (○) *M. smegmatis* mc^2^155 carrying pCG76 at 30°C; (•) *M. smegmatis* mc^2^155 carrying pCG76 at 42°C. B. Growth curves of *M. smegmatis* LS2 after shifting temperature from 30°C to 42°C. *M. smegmatis* LS2 was grown at 30°C to 0.10 OD_600_, and then transferred to a 42°C incubator. The culture grown at 30°C was as control. The OD_600_ was determined at the interval of 24 hours. X-axis showed the time point after temperature transferred. (▪) *M. smegmatis* LS2 was grown at 30°C for 24 h, and then grown at 42°C; (□) *M. smegmatis* LS2 kept at 30°C was a control. C. CFU of *M. smegmatis* LS2 at 30°C and 42°C. CFU determinations were taken at the same time points as (B). Dilutions were spread on LB agar plates and the plates were incubated at 30°C before CFU were counted. Black bars were *M. smegmatis* LS2 at 30°C; White bars were *M. smegmatis* LS2 which grown at 30°C for 24 h and then grown at 42°C.

Growth curves of *M. smegmatis* LS2 and *M. smegmatis* mc^2^155 carrying pCG76 at 30°C and 42°C were obtained to determine the essentiality of MSMEG_1556 for the growth of mycobacteria. The results showed that *M. smegmatis* LS2 was only able to grow at 30°C but not at 42°C, whereas *M. smegmatis* mc^2^155 carrying pCG76 was able to grow at both 30°C and 42°C (Fig. 6A), which was consistent to the growth curve of wild type *M. smegmatis* mc^2^155 (data not shown). To measure CFU, *M. smegmatis* LS2 was grown at 30°C to 0.10 OD_600_, and then the culture was switched to an incubator at 42°C. The OD_600_ and CFU over time were obtained as shown (Fig. 6B, 6C). Since Rv3441c protein was produced in *M. smegmatis* LS2 cells at 30°C, the cells multiplied for the first 24 hours after the temperature shift. Then dramatically, with increased incubation time at 42°C, the CFUs dropped as the amount of Rv3441c protein decreased. These results demonstrated that MSMEG_1556 gene is essential in *M. smegmatis*.

### Rv3441c protein was not detected in *M. smegmatis* LS2 strain

**Figure 7:**
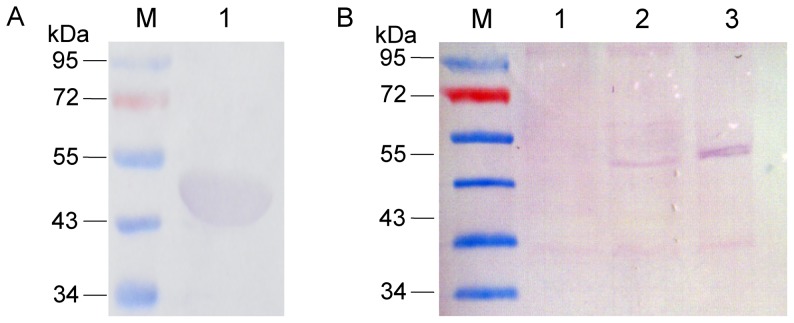
Western blot analysis of proteins from *M. smegmatis* mc^2^155, *M. smegmatis* LS2 at 30°C and *M. smegmatis* LS2 at 42°C. A. The purified Mtb Rv3441c protein was detected by using (anti)-MSMEG_1556 antibody. The result showed (anti)-MSMEG_1556 antibody had a cross reaction with Rv3441c. M. PageRuler prestained protein ladde; lane 1. the purified Mtb Rv3441c protein. B. Rv3441c protein in *M. smegmatis* LS2 grown at 42°C and 30°C and MSMEG_1556 protein in *M. smegmatis* mc^2^155 strain were analyzed by using (anti)-MSMEG_1556 antibody. M. PageRuler prestained protein ladder; lane 1. the Rv3441c protein in *M. smegmatis* LS2 grown at 42°C was not detectable; lane 2. the Rv3441c protein in *M. smegmatis* LS2 grown at 30°C was detectable; lane 3. the MSMEG_1556 protein in *M. smegmatis* mc^2^155 strain was detectable.

Western blot was used to analyze the expression levels of Rv3441c protein. (Anti)-MSMEG_1556 antibody performed a cross reaction with Rv3441c protein (Fig. 7A), demonstrated that (anti)-MSMEG_1556 antibody could be used for detect the Rv3441c protein in *M. smegmatis* LS2 strain. Western blot results showed that when *M. smegmatis* LS2 was grown at 42°C, the expression of Rv3441c protein was not detected compared to the Rv3441c protein in *M. smegmatis* LS2 grown at 30°C (Fig. 7B). The MSMEG_1556 in wild type *M. smegmatis* was also detected. The data indicated that lacking Rv3441c protein in *M. smegmatis* LS2 had effect on bacterial growth at 42°C.

### *M. smegmatis* LS2 grown at 42°C had a morphological change

**Figure 8:**
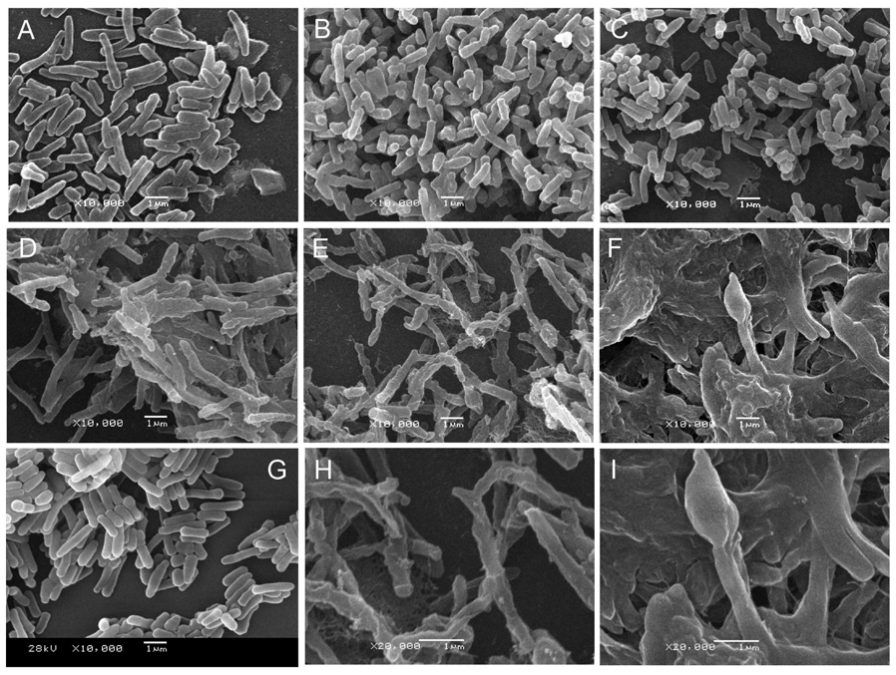
Scanning electron micrographs of *M. smegmatis* LS2 strain after shifting temperature from 30°C to 42°C. *M. smegmatis* LS2 grown at 30°C was switched to 42°C when OD_600_ reached to 0.01, and *M. smegmatis* LS2 strain kept growing at 30°C was as control. The cells were harvested after temperature switched for 72 h, 120 h and 192 h, respectively. The micrographs showed that shape and size of *M. smegmatis* LS2 strain kept growing at 30°C (A, B, C) were similar to those of wild type strains, whereas the *M. smegmatis* LS2 strain at 42°C became longer shapes, “bulb” heads, rougher cell surface and even lysis (D, E, F). A, B, C. *M. smegmatis* LS2 grown at 30°C for 72, 120, 192 h (10000×); D, E, F. *M. smegmatis* LS2 grown at 42°C for 72, 120 and 192 h (10000×). H, I. *M. smegmatis* LS2 grown at 42°C for 120 and 192 h (20000×); G. Wild type *M. smegmatis* mc^2^155 (10000×).

The morphology of *M. smegmatis* mc^2^155, *M. smegmatis* LS2 grown at 30°C and *M. smegmatis* LS2 grown at 42°C were observed by using SEM. The results showed that the cellular shape and size of *M. smegmatis* LS2 grown at 42°C (switched from 30°C) changed compared to those kept growing at 30°C and wild type *M. smegmatis* mc^2^155. As the incubation time extended, *M. smegmatis* LS2 strain at 42°C became longer shapes, “bulb” heads, rougher cell surface and even lysis (Fig. 8D, 8E, 8F). Whereas, the shape and size of *M. smegmatis* LS2 strain kept growing at 30°C (Fig. 8A, 8B, 8C) were similar to those of wild type *M. smegmatis* mc^2^155 (Fig. 8G).

## Discussion

UDP-GlcNAc is an essential common precursor of bacterial cell wall PG and outer membrane lipopolysaccharide, as well as important for the synthesis of the enterobacterial common antigen [13], [14]. The pathway for UDP-GlcNAc synthesis in prokaryotes is somewhat different from that in eukaryotes [17]. Three enzymes, glutamine fructose-6-phosphate transferase (GlmS), phosphoglucosamine mutase (GlmM) and glucosamine-1-phosphate acetyl transferase/N-Acetylglucosamine-1-phosphate urididyl transferase (GlmU), catalyzed the formation of UDP-GlcNAc in *E. coli* have been identified [13], [15], [16], [17], [18]. GlmM catalyzes the conversion of GlcNH_2_-6-P to GlcNH_2_-1-P. The homologs of GlmM were also found in other Gram-negative bacteria e.g. *H. influenzae, H. pylori, P. aeruginosa* [27], [28], [29] and Gram-positive bacteria e.g. *S. gordonii* and *S. aureus* [30], [31]. The *glmM* gene was essential for cell growth in Gram-negative bacteria (e.g. *E. coli*). The inactivation of *glmM* gene in *E. coli* was followed by various alterations of cell shape and finally cells were lysed [13]. This was most probably due to the progressive depletion of precursors for PG and lipopolysaccharide synthesis. However, the mutation of *glmM* gene only reduced the growth rate and increase cell autolysis in Gram-postive bacteria [30], [32]. Furthermore, the *glmM* mutation also reduced the formation of bacterial biofilm and increased sensitivity to penicillins [30], [32].

In mycobacteria, UDP-GlcNAc is an important sugar donor for both formation of dissarchride linker and biosynthesis of peptidoglycan in mycobacterial cell wall, therefore, lacking UDP-GlcNAc could have effect on the structural integration of mycobacterial cell wall and further on their cell morphology. Ec GlmM has been well characterized as phosphoglucosamine mutase to catalyze the second step in the synthesis of *E. coli* UDP-GlcNAc [13], and Msm MSMEG_1556 protein and Mtb Rv3441c protein have significant homology to Ec GlmM.

To detect the phosphoglucosamine mutase activity of Msm MSMEG_1556 protein and Mtb Rv3441c protein, it is required to acquire soluble protein. Mtb Rv3441c protein was produced by using pET16b vector, unfortunately, Mtb Rv3441c protein was insoluble (data not shown). To avoid the formation of inclusion bodies, a cold-shock expression vector pCold II with the Rv3441c gene was constructed. This vector was designed to perform efficient protein expression utilizing promoter derived from *cspA* gene, which was one of the cold-shock gene. When the incubation temperature of *E. coli* host cells was reduced sufficiently, the growth is temporarily halted and almost of protein expression decrease, while expression of a group of proteins called “cold-shock proteins” was specifically induced. A significant overproduction of soluble Mtb Rv3441c protein in *E. coli* BL21(DE3) was observed. The Msm MSMEG_1556 protein was also produced by using the same protocol.

In our study, we also set up an enzyme assay for detection of phosphoglucosamine mutase activity. DNTB chromogenic method was more convenient and time saving, which facilitates high-throughput inhibitor screening, compared with previous methods by autoradiography and HPLC [13], [17].

The activity of both Msm MSMEG_1556 protein and Mtb Rv3441c protein was dependent on the presence of Mg^2+^. Glc-1,6-diP was also required for activity. The purified proteins exhibited little activity without Glc-1,6-diP, however, phosphoglucosamine mutase activity was remarkably enhanced in the presence of this compound. The phosphorylated phosphoglucosamine mutase was assumed to be active [13]. It is unclear that Glc-1,6-diP is a phosphorylating agent or activator. We attempt to co-crystallize Glc-1,6-diP and Mtb Rv3441c protein so as to reveal this mechanism in follow-up research.

Mtb Rv3441c gene (annotated as *mrsA* in TubercuList Server) has been proved to be essential for the growth of cells by using Himar1-based transposon site hybridization (TraSH) methodology [33]. To assess the effect of mutated *glmM* gene on cell growth, morphology, cell wall structure, etc., we used a model mycobacterial strain, *M. smegmatis*, to construct conditional MSMEG_1556 gene knockout strain LS2 by inserting *kan^R^* cassette. *M. smegmatis* LS2 was unable to grow at 42°C (non-permissive temperature) since the rescue plasmid carrying Mtb Rv3441c gene could not replicate. It demonstrated that the MSMEG_1556 gene is essential for the growth of *M. smegmatis*. Furthermore, we found that GlmM protein was not expressed in *M. smegmatis* LS2 strain. To observe morphological change of LS2 strain, the LS2 cells were harvested after shifting temperature from 30°C (permissive temperature) to 42°C. The morphological analysis of LS2 by SEM revealed that the LS2 cells were longer and had rougher surface compared to the wild type cells. With the increased incubation time at 42°C, many cells fused and lysed eventually. These results suggested that lacking GlmM could block the PG synthesis and make the structure of cell wall changed, resulting in cell death. Therefore, GlmM is a potential target for development of anti-tuberculosis drugs.

## Supporting Information

Table S1 Bacterial strains and plasmids used in this study.
(DOC)
