# Supplementary material for: Identification of M. tuberculosis Rv3441c and M. smegmatis MSMEG_1556 and Essentiality of M. smegmatis MSMEG_1556
Source: PLoS One. 2012 Aug 8;7(8):e42769. doi: 10.1371/journal.pone.0042769 (PMC3414508; doi:10.1371/journal.pone.0042769)
Supplement: Table S1 — Bacterial strains and plasmids used in this study. (DOC) [file pone.0042769.s001.doc]

**Table S1. Bacterial strains and plasmids used in this study**.

| **Strains/ plasmids** | Description | Source |
| --- | --- | --- |
| **Strains** |  |  |
| *E.coli* DH5α | Used for cloning and propagation of plasmids | Invitrogen |
| *E. coli* NovaBlue | Used for cloning and propagation of plasmids | Novagen |
| *E.coli* BL21(DE3) | Used as a DNA template to amplify Ec *glmM* and for expressing proteins | Novagen |
| *M. tuberculosis* H37Rv | Used as a DNA template to amplify Mtb Rv3441c gene | Colorado State University |
| *M. smegmatis* mc^2^155 | Used as a DNA template to amplify Msm MSMEG_1556 gene and its upstream sequence (500 bp) and used for allelic exchange experiments | ATCC |
| *M. smegmatis* LS1 | *M. smegmatis* mc^2^155 with pLS5 plasmid integrated into the MSMEG_1556 gene locus | This study |
| *M. smegmatis* LS2 (mcΔ1556::pLS7) | MSMEG_1556 gene knock out strain carrying pLS7 plasmid | This study |
| **plasmids** |  |  |
| pJET1.2 | Carries *amp^R^* gene and *eco47IR* lethal gene; used for PCR product with blunt end | Fermentas |
| pMD18-T | Carries *amp^R^* gene; used for cloning PCR product with A’ at 3’ ends | Takara |
| pET16b | Carries *amp^R^* gene; used for expression Ec GlmM protein | Novagen |
| pCold II | Carries *amp^R^* gene; used for expression Msm MSMEG_1556 protein and Mtb Rv3441c protein | Takara |
| pUC4K | Carries *amp^R^* gene and *kan^R^* cassette | GE Healthcare |
| pPR27-*xylE* | Carries *gen^R^*, *sacB* and *xylE* genes; carries replication origin for *E. coli* and temperature-sensitive replication origin for mycobacteria | [11] |
| pET23b-Phsp60 | Carries *amp^R^* gene; carries *M. bovis* BCG hsp60 promoter | [11] |
| pCG76 | Carries *str^R^* gene; carries replication origin for *E. coli* and temperature-sensitive replication origin for mycobacteria. | [25] |
| pKJ1 (pJET-Msm MSMEG_1556) | Msm MSMEG_1556 gene was cloned to the EcoRV site of pJET1.2/blunt vector | This work |
| pKJ2 (pCold II-MSMEG_1556) | Msm MSMEG_1556 gene in pKJ1 was cloned to the NdeI and EcoRI sites of pCold II | This work |
| pKJ3 (pJET-Ec *glmM*) | Ec *glmM* was cloned to the EcoRV site of pJET1.2/blunt vector | This work |
| pKJ4 (pET16b-Ec *glmM*) | Ec *glmM* in pKJ3 was cloned to the NcoI and BamHI sites of pET16b | This work |
| pLS1 (pMD18-Mtb Rv3441c) | Mtb Rv3441c gene was cloned to the EcoRV site of pMD18-T | This work |
| pLS2 (pCold II-Mtb Rv3441c) | Mtb Rv3441c gene in pLS1 was cloned to the NdeI and BamHI sites of pCold II | This work |
| pLS3 (pMD18- Msm MSMEG_1556) | Msm MSMEG_1556 gene and its upstream sequence was cloned to the EcoRV site of pMD18-T | This work |
| pLS4 (pMD18-Msm MSMEG_1556::*kan^R^* ) | The *kan^R^* cassette was inserted to the XhoI site of Msm MSMEG_1556 gene in pLS3 | This work |
| pLS5 (pPR27-Msm MSMEG_1556::*kan^R^*) | Conditional replication plasmid, Msm MSMEG_1556 gene::*kan^R^* in pLS4 was cloned to the SpeI and NotI sites of pPR27-*xylE* | This work |
| pLS6 (pET23b-Phsp60-Mtb Rv3441c) | Mtb Rv3441c gene in pLS1 was cloned to the NdeI and BamHI sites of pET23b-Phsp60 | This work |
| pLS7 (pCG76-Phsp60- Mtb Rv3441c) | Rescue plasmid, Phsp60- Mtb Rv3441c gene in pLS6 was cloned to the XbaI and BamHI sites of pCG76 | This work |
